# Assessment of the application of the FA280—a fully automated fecal analyzer for diagnosing clonorchiasis: a mixed-method study

**DOI:** 10.1186/s40249-024-01271-8

**Published:** 2025-01-06

**Authors:** Si-Yue Huang, Qing-Sheng Zeng, Xin-Fu Shi, Yun-Ting He, Yue-Yi Fang, Ying-Si Lai

**Affiliations:** 1 Department of Medical Statistics, School of Public Health, Sun Yat-sen University, Guangzhou, Guangdong People’s Republic of China; 2 Xinhui District Center for Disease Control and Prevention, Jiangmen, Guangdong People’s Republic of China; 3 Institute of Parasitic Diseases, Guangdong Provincial Center for Disease Control and Prevention, Guangzhou, Guangdong People’s Republic of China; 4 Sun Yat-sen Global Health Institute, Sun Yat-sen University, Guangzhou, Guangdong People’s Republic of China; 5 Health Information Research Center, Guangdong Key Laboratory of Medicine, School of Public Health, Sun Yat-sen University, Guangzhou, Guangdong People’s Republic of China; 6 Guangzhou Joint Research Center for Disease Surveillance, Early Warning and Risk Assessment, Guangzhou, Guangdong People’s Republic of China

**Keywords:** FA280 fecal analyzer, Application evaluation, *Clonorchis sinensis*, Diagnosis, Mixed-method

## Abstract

**Background:**

Clonorchiasis is an important foodborne parasitic disease in China caused by *Clonorchis sinensis*. Accurate and rapid diagnosis of this disease is vital for treatment and control. Traditional fecal examination methods, such as the Kato-Katz (KK) method, are labor-intensive, time-consuming, and have limited acceptance. The FA280, an advanced automated fecal analyzer, increases efficiency while significantly reducing labor load. This study aims to evaluate its performance, applicability, and scalability in clonorchiasis diagnosis to explore its potential application in the future.

**Methods:**

A mixed-methods study integrating both quantitative and qualitative approaches was conducted. The quantitative component consisted of a cross-sectional survey in Xinhui District, Guangdong, China, to evaluate the diagnostic performance of the FA280. The positive rate and agreement between the FA280 and the KK method were evaluated using McNemar’s test. Additionally, Pearson’s Chi-square test was used to analyze the consistency of positive results between the two methods across various eggs per gram (EPG) groups under different cut-off values. The qualitative component included semi-structured individual interviews with medical staff and institutional administrators to examine the FA280’s applicability and potential for broader adoption, with thematic analysis of the data.

**Results:**

In the quantitative study of 1000 participants, both the FA280 and KK methods detected clonorchiasis with a positive rate of 10.0%, achieving 96.8% agreement and showing no significant difference (*P* > 0.999). The kappa value was 0.82 (95% confidence interval: 0.76–0.88), indicating a strong agreement between the methods. The agreement rate for positive results between the two methods was significantly higher in the high infection intensity group compared to the low infection intensity group (*P* < 0.05). The qualitative study, which involved interviews with three medical staff and two administrators revealed that the FA280 outperformed the KK method in testing procedures, detection results, and user acceptance. The benefits, challenges, and suggestions of FA280 promotion were also emphasized.

**Conclusions:**

This study demonstrated the FA280’s application value in clonorchiasis diagnosis by assessing its detection performance, applicability, and scalability. These findings contribute to the future prevention and control of the disease.

**Graphical Abstract:**

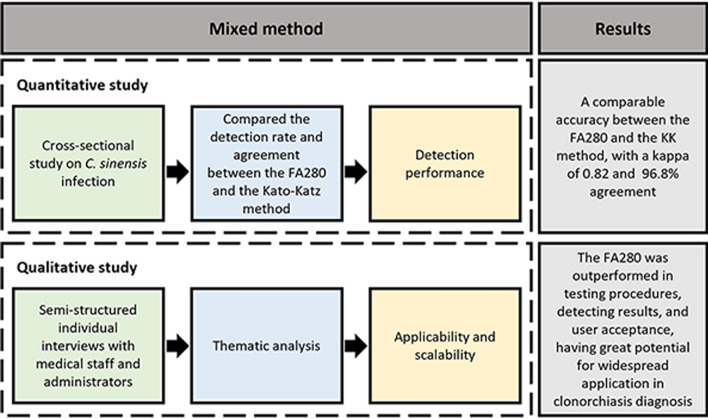

**Supplementary Information:**

The online version contains supplementary material available at 10.1186/s40249-024-01271-8.

## Background

Clonorchiasis is an important foodborne parasitic disease caused by *Clonorchis sinensis*, primarily transmitted through the consumption of raw or undercooked freshwater fish (mostly of the Cyprinidae family) containing metacercaria [1, 2]. China accounts for over 82% of global cases with approximately 10.82 million infections, predominantly in the southeastern parts covering Guangdong province and Guangxi Zhuang Autonomous Region, as well as the northeastern provinces of Heilongjiang and Jilin [3–7]. *C. sinensis* infection damages the hepatobiliary system, potentially leading to cholangitis, cholecystitis, gallstone, and cholangiocarcinoma, which contribute to a significant disease burden [8–10]. However, as there is no obvious clinical symptom at the early stage of the infection, early diagnosis plays a critical role in treatment and control [11, 12]. Current control strategies for clonorchiasis in China include chemotherapy, health education and promotion, and environmental reconstruction [13, 14]. In highly endemic areas, preventive chemotherapy is often conducted without prior disease detection, which affects medication adherence and reduces its effectiveness [15]. Therefore, a definitive diagnosis is essential for successful control work. On the one hand, diagnosis can guide the chemotherapy precisely to infected individuals, thus improving compliance with medications among the public [15, 16]. On the other hand, the diagnosis assists the evaluation of local endemicity, subsequently influencing the selection of intervention measures and the assessment of their effectiveness [15].

At present, the gold standard for diagnosis of clonorchiasis involves detecting eggs in feces, but there is no gold standard method for fecal examinations [17]. The Kato-Katz (KK) and the formalin-ether concentration technique (FECT) are commonly used and have relatively high sensitivity [18, 19]. However, these methods differ significantly in their applications. The KK method is widely used in large-scale epidemiological surveys, drug efficacy evaluations, and intervention monitoring and assessments in China [20–23]. In contrast, the FECT’s sensitivity is limited by sample insufficiency, and its complex centrifugation steps make it impractical for mass screening [24, 25]. Moreover, both methods are labor-intensive, monotonous, time-consuming, and heavily reliant on the expertise and skills of trained microscopists. Additionally, medical personnel often exhibit reluctance to handle fecal matter. Hence, new diagnostic tools with lower complexity, higher throughput, and reduced costs are urgently needed to improve clonorchiasis diagnosis.

Automated fecal analyzers have emerged as promising tools for the diagnosis of parasitic infections, offering rapid and convenient fecal examination through automated egg identification and imaging. Despite these advances, only a few studies have explored their application in detecting *C. sinensis* infection [26–29]. For instance, analyzers such as the AVE-562 (AVE Science & Technology Co., Ltd., Changsha, Hunan, China) and KU-F20 (Zhuhai Keyu Biological Engineering Co., Ltd., Zhuhai, Guangdong, China) have been evaluated for their effectiveness in identifying *C. sinensis* eggs but demonstrated suboptimal accuracy and agreement with traditional methods [26, 27]. The FA280 (Sichuan Orienter Bioengineering Co., Ltd., Chengdu, Sichuan, China), a newly automatic digital fecal analyzer, has shown potential for greater accuracy and improved performance through innovations such as intelligent sample dilution, high-frequency pneumatic mixing, AI-driven parasite egg identification, and high-resolution imaging [30, 31]. Preliminary studies have shown the FA280’s excellent capability in differentiating various parasites, including soil-transmitted helminths and *Taenia* spp., and has demonstrated its comparable performance to FECT and enzyme-linked immunosorbent assay (ELISA) in detecting *C. sinensis* [28, 29].

Nonetheless, several significant challenges persist. Previous studies have lacked direct comparisons between the FA280 and the KK method, which is a commonly used and reliable diagnostic technique for large-scale clonorchiasis surveys in China [24, 25]. Furthermore, existing research has been limited to hospital-based patients, making it impractical for community-based screening, the primary way for discovering infection cases [32]. Moreover, previous studies have predominantly focused on quantitative analyses, which provide statistical insights but often overlook crucial contextual and subjective dimensions [33, 34]. Incorporating qualitative approaches can provide a more comprehensive understanding of user experiences, social factors, and the interplay between knowledge and action, thus addressing the gaps left by quantitative studies [33–35].

Therefore, our study employed a mixed-method study integrating both quantitative and qualitative approaches. Using the KK method as the reference and focusing on a community-based population, we assessed the performance, applicability, and scalability of the FA280 in diagnosing clonorchiasis, thus comprehensively exploring its application value in the future.

## Methods

A mixed-method design was used to collect quantitative and qualitative data. The quantitative study of this study aimed to evaluate the detection performance of the FA280. The qualitative study aimed further to elucidate its applicability and potential for future promotion.

### Quantitative study

#### Study design and study area

The quantitative study used a cross-sectional study design to investigate *C. sinensis* infection, conducted from August to September 2023 in Xinhui District, Jiangmen City, Guangdong Province, China. Xinhui District, located in the southwestern part of the Pearl River Delta, occupies the south-central region of Guangdong Province. It covers an area of 1354 km^2^ and a population of 909,277 as of 2020. The district lies between 22°05′–22°35′ north latitude and 112°47′–113°15′ east longitude, with a subtropical marine monsoon climate [36]. Known for its aquaculture and tradition of consuming raw freshwater fish, Xinhui District is a significant endemic area of clonorchiasis. Since 2016, it has been designated as a provincial surveillance site for the disease [37].

#### Sample size

The required sample size for the agreement test was calculated using PASS software (version 2021; NCSS LLC, Kaysville, Utah, USA). The calculation, based on ${\kappa }_{1}$ = 0.9 [29], ${\kappa }_{0}$ = 0.8, a 95% confidence level, and 90% statistical power, resulted in 689 subjects. Considering a 30% dropout rate, we gathered 1000 participants for the study.

#### Sample collection

Participants were included via a multi-stage cluster sampling method. Five towns by geographic locations of east, west, north, south, and middle were randomly selected. One village from each town and 200 participants per village were randomly selected, totaling 1000 people for stool examination. Collection boxes were delivered a day before collection.

#### Detection of *C. sinensis* infections

Each participant was asked to provide one stool sample. Then both the FA280 fully automated fecal analyzer and the KK method were used for the detection of *C. sinensis*.

#### *Detection by the FA280*

**Fig. 1:**
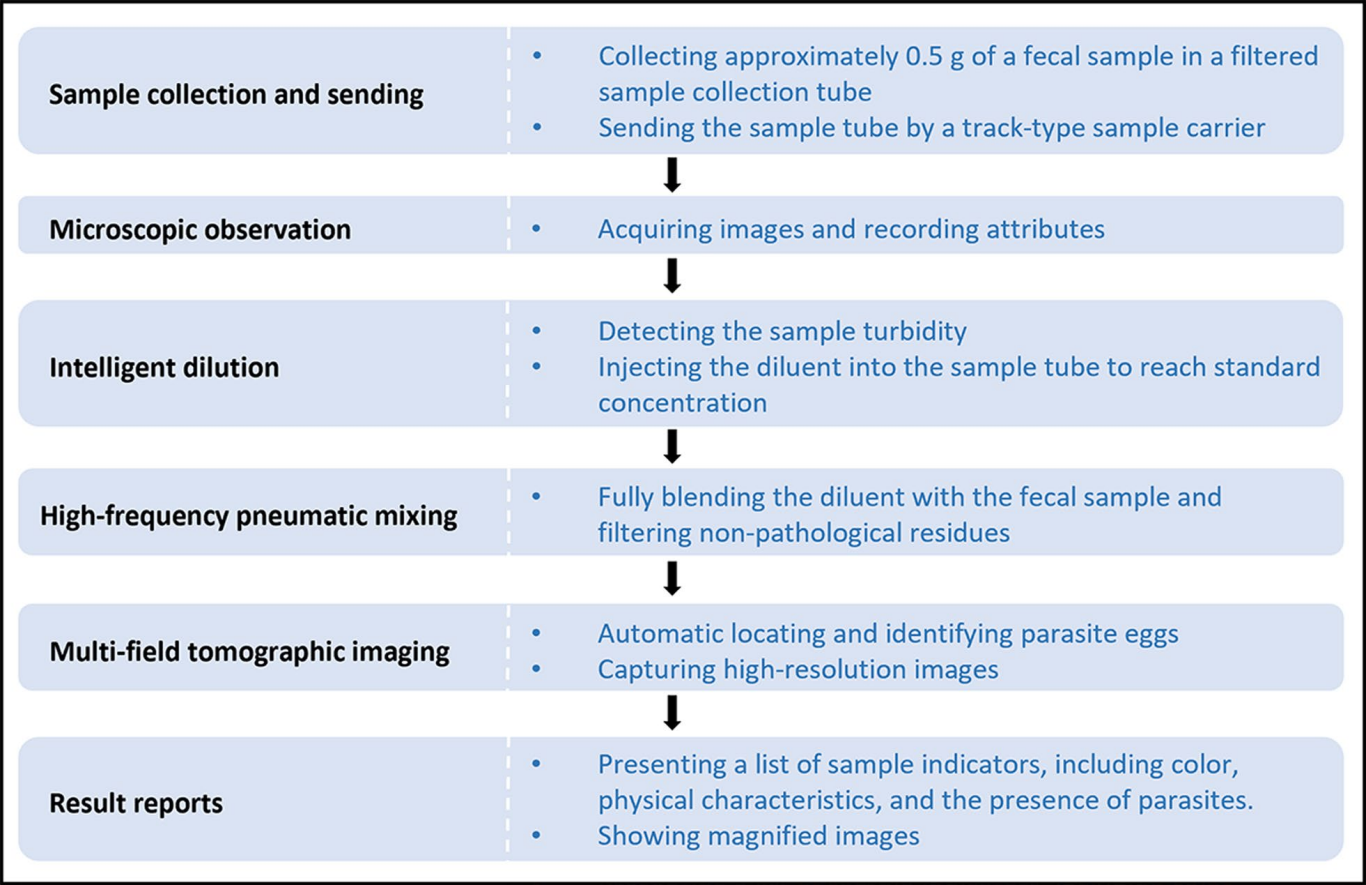
The operation procedures of the FA280

The FA280 fully automated fecal analyzer employed automatic sedimentation and concentration technology for detection. Approximately 0.5 g of a fecal sample was collected in a filtered sample collection tube and submitted for testing [38]. The device initiated microscopic observation, acquiring images and recording attributes of (or including) color, shape, and consistency. After the diluent was added and mixed, the microscope of the FA280 automatically focused and captured high-resolution images through multi-field tomography. The images were subsequently analyzed by the software to generate a report. The operational procedures of the FA280 were shown in Fig. 1, and more details were provided in Additional file 3.

#### *Detection by the KK method*

Two smears were prepared per fecal sample. For each smear, 41.7 mg of sieved stool was transferred in a plastic template on a glass slide, covered with cellophane soaked in glycerol and malachite green. Four experienced technicians examined smears under a microscope (CX-23, Olympus Corporation, Japan), counting *C. sinensis* eggs. Ten stool samples from each study village were re-examined by a professional staff to conduct quality control.

#### Statistical analysis

Statistical analyses were conducted using R software (version 4.0.3; R Foundation for Statistical Computing, Vienna, Austria). McNemar’s test was employed to compare the detection of parasite-positive samples by the FA280 and the KK method, with a *P*-value < 0.05 considered significant. The kappa (*κ*) statistic with a 95% confidence interval (*CI*), was used to evaluate the agreement between the two techniques. The $\kappa$ values of 0–0.20, 0.21–0.40, 0.41–0.60, 0.61–0.80, and 0.81–1.00 indicated poor, fair, moderate, good, and almost perfect agreement, respectively [39]. The infection intensity, measured as eggs per gram (EPG) of feces, was calculated by multiplying the arithmetic mean of the egg counts from two KK smears per sample by 24. Those who had *C. sinensis* eggs detected by either of the two fecal examination methods were defined as positive individuals. To explore the relationship between the consistency of positive results from the two methods and the infection intensity, we classified the EPG obtained from the KK method (hereafter referred to as EPG) of positive individuals with different cut-off values and different group counts. The proportion of consistent positive results was calculated for different EPG groups and Pearson’s Chi-square test was applied to assess the difference.

### Qualitative study

#### Study design

The qualitative study used semi-structured individual interviews. The inclusion criteria required participants to have experience with both the FA280 and the KK methods. A purposive sampling method was used to recruit participants. Three health service staff were interviewed to evaluate FA280’s applicability, and two medical institution administrators were interviewed to discuss its potential for widespread adoption. Interviews were conducted individually and followed the principle of information saturation, meaning that the interview continued until no new information emerged [40]. Regular discussions with colleagues were held to review sampling decisions and data collection, ensuring the data was comprehensive and accurate.

#### Data collection

The interview guidelines (Additional file 1) for the qualitative study were developed based on prior research and advice from two epidemiological experts. The interviewer received comprehensive training on interview techniques, ethical considerations, and the management of sensitive topics before the interviews. Each interview was conducted online via the Tencent Meeting between February and March 2024, lasting approximately 10–20 min. With permission, the interviews were audio recorded, and the recordings were immediately reviewed for clarity and completeness after each interview. The audio recordings were transcribed verbatim by a professional transcribing service and anonymized before analysis.

#### Data analysis

Thematic analysis, as outlined by Braun and Clarke, was utilized [41]. Researchers read and re-read each transcript to be familiar with the interview data. The open coding was undertaken to identify meaningful words related to the study aim from each transcript, and the research team verified the coding to prevent bias. All codes were then grouped based on their similarities, and potential sub-themes and themes were created. Finally, these sub-themes and themes were reviewed to ensure consistency between the results and the data (Additional file 2).

### Ethical considerations

This study was approved by the ethics committee of Sun Yat-sen University (Approval no. 2021–087). The study purpose and methods were clearly explained to the participants, whose anonymity and confidentiality were maintained. All participants who were diagnosed positive for *C. sinensis* were provided with free chemotherapy treatment.

## Results

### Quantitative results

#### Comparison of the FA280 and the KK for parasite detection

**Fig. 2:**
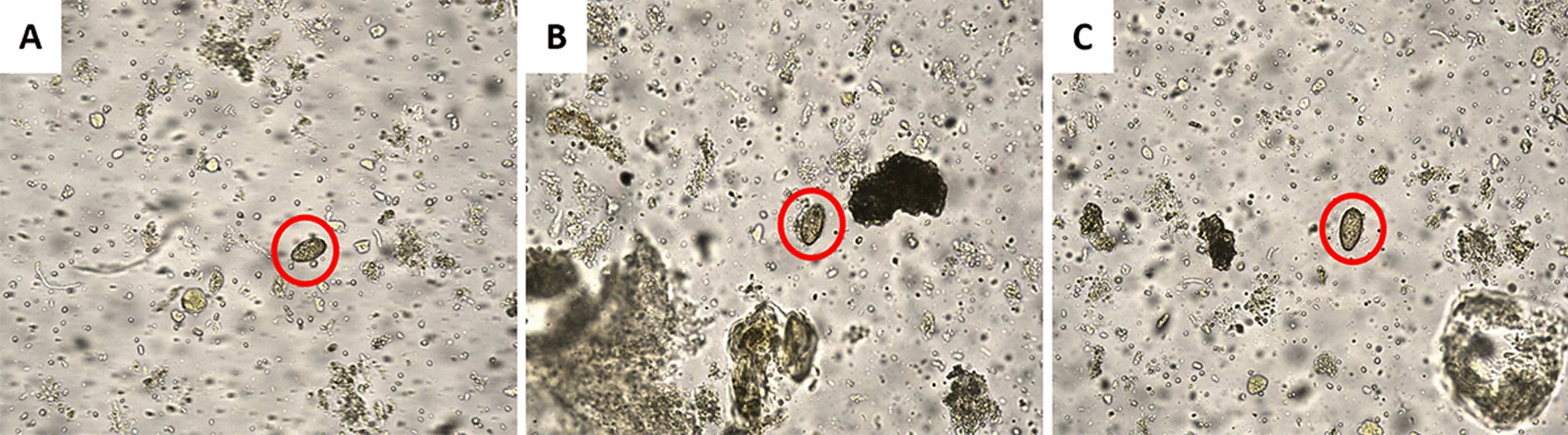
High-resolution images from the FA280. **A**–**C **were *C. sinensis* eggs (red circles)

A total of 1000 participants provided stool samples, including 478 (47.8%) males and 522 (52.2%) females. The age distribution was as follows: < 20 years (17.3%), 20–39 years (20.9%), 40–59 years (29.2%), and ≥ 60 years (32.6%). The overall average EPG was 12.35. Males (13.6%) had a higher positive rate than females (6.7%). Participants in the 40–59 age group had the highest positive rate (14.0%), while the < 20 age group had the lowest positive rate (6.4%). No statistically significant differences in positive rates between the two methods were observed across different age and gender groups (Pearson’s Chi-square test, *P* > 0.05) (Table 1). The FA280 and the KK method both showed a 10.0% overall positive rate for *C. sinensis*. There was no statistically significant difference between the two methods (McNemar’s test, *P* > 0.999) (Table 2). The overall agreement rate was 96.8%, with highly acceptable agreement ($\kappa$ = 0.82, 95% *CI:* 0.76–0.88). Figure 2 showed high-resolution images with 5 million pixels from the FA280 digital microscope depicting eggs of *C. sinensis*. The figures regarding the collection tube, sample detection, and results output of the FA280 could be found in Additional file 3.

**Table 1:** The demographic characteristics and infection details of the study subjects

		No. of individuals/proportion (%)	Average EPG from KK method	Detection from KK method/rate (%)	Detection from FA280/ rate (%)	*P*
Gender						
Female		522 (52.2)	11.0	60 (7.7)	65 (6.7)	0.302
Male		478 (47.8)	13.8	40 (12.6)	35 (13.6)	0.332
Age (years)						
< 20		173 (17.3)	19.2	10 (5.8)	11 (6.4)	> 0.999
20–39		209 (20.9)	12.6	25 (12.0)	22 (10.5)	0.505
40–59		292 (29.2)	10.2	37 (12.7)	41 (14.0)	0.453
≥ 60		326 (32.6)	10.5	37 (8.6)	26 (8.00)	0.683
Total		1000 (100.0)	12.3	100 (10.0)	100 (10.0)	> 0.999

*EPG* eggs per gram of feces, *KK* Kato-Katz

**Table 2:** Results of the FA280 and the Kato-Katz method for *C. sinensis* infection diagnosis

		Kato-Katz		Total
		No. positive	No. negative	
FA280	No. positive	84	16	100
	No. negative	16	884	900
Total		100	900	1000

#### Relationship between the consistency of positive results and the infection intensity

The agreement rate of positive results between the FA280 and the KK method in the group with low infection intensity was significantly higher than that in group with high infection intensity (Pearson’s Chi-square test, *P* < 0.05), regardless of the chosen cut-off values and group counts (Table 3).

**Table 3:** The consistency of positive results between the FA280 and the Kato-Katz method across different infection intensity groups under different cut-off values

EPG from KK method	No. of consistently positive cases	No. of positive individuals	Agreement rate (%)	*P*
Divided into two groups				
< 24	8	28	28.6 (11.8, 45.3)	< 0.001
≥ 24	76	88	86.4 (79.2, 95.5)	
< 48	32	54	59.3 (46.2, 72.4)	0.003
≥ 48	52	62	83.9 (74.7, 95.0)	
< 72	52	79	65.8 (55.4, 76.3)	0.020
≥ 72	32	37	86.5 (75.5, 97.5)	
< 96	55	83	66.3 (56.1. 76.4)	0.019
≥ 96	29	33	87.9 (76.7, 99.0)	
< 120	58	88	65.9 (56.0, 75.8)	0.005
≥ 120	26	28	92.9 (83.3, 100.0)	
< 144	59	90	65.6 (55.7, 75.4)	0.002
≥ 144	25	26	96.2 (88.8, 100.0)	
< 168	63	94	67.0 (57.5, 76.5)	0.007
≥ 168	21	22	95.5 (86.8, 100.0)	
≥ 192	68	100	68.0 (58.9, 77.1)	0.018
≥ 192	16	16	100.0 (100.0, 100.0)	
< 216	69	101	68.3 (59.2, 77.4)	0.024
≥ 216	15	15	100.0 (100.0, 100.0)	
< 240	70	102	68.6 (59.6, 77.6)	0.032
≥ 240	14	14	100.0 (100.0, 100.0)	
Divided into two groups				
< 24^#*^	8	28	28.6 (11.8, 45.3)	< 0.001
24–95^#^	47	55	85.5 (76.1, 94.8)	
≥ 96^*^	29	33	87.9 (76.7, 99.0)	
< 48^*^	32	54	59.3 (46.2, 72.4)	0.002
48–143	27	36	75.0 (60.9, 89.1)	
≥ 144^*^	25	26	96.2 (88.8, 100.0)	
< 72^*^	52	79	65.8 (55.4, 76.3)	0.019
72–191	16	21	76.2 (58.0, 94.4)	
≥ 192^*^	16	16	100.0 (100.0, 100.0)	

^*^/^#^ The difference was statistically significant in multiple comparisons, *EPG* eggs per gram of feces, *KK* Kato-Katz

### Qualitative results

#### Findings on differences between the FA280 and the KK methods

Three health service staff participated in this study. All participants were female. The average years of working experience for control and fecal examination of clonorchiasis was 20.3 years. Demographic details of interviews are provided in Additional file 2.

Differences between the FA280 and the KK methods were analyzed thematically around (Table 4): (i) differences in testing procedures; (ii) differences in detecting results; and (iii) differences in acceptance

**Table 4:** Overview of themes and subthemes relative to differences between the FA280 and the Kato-Katz method

Theme	Subtheme
Differences in testing procedures	Feeling about the testing process
	Laboratory environment
	Time requirement
	Smear examination
	Requirement for technicians
Differences in detecting results	Accuracy
	Involvement human factor
	Infection intensity
Differences in acceptance	Acceptability
	Future choice

Theme 1: differences in testing procedures.

#### Feeling about the testing process

All participants considered the KK method’s testing process to be complex and cumbersome, although it was relatively easy to learn. However, they pointed out that the FA280 was straightforward, and easier to master than the KK method for detecting *C. sinensis*.

*“The KK method is a bit cumbersome to operate.”*

*“The KK method is easy to learn.”*

*“The operation of the fecal analyzer is quite simple. Once the samples are collected, you place them directly into the instrument and let it do the rest.”*

*“Learning to use the fecal analyzer is much easier compared to the KK method.”*

#### Laboratory environment

Every participant mentioned that the KK method created a dirty and smelly environment. In contrast, the FA280 had less odor and one participant emphasized that the operating environment for the fecal analyzer was very sanitary.

*“During the preparation of KK smears, there is an odor, and the environment is not pleasant, quite dirty, and smelly. Then, during the process of examining the smears, as it involves using a microscope and looking at them with the naked eye, there is still an odor, and the environment is not pleasant either.”*

*“Both the fecal analyzer and the KK method have odors, so how could there be no smell? However, the KK method has a stronger odor, while the instrument has less odor.”*

*“The environment is excellent, and using fecal analyzer is very clean.”*

#### Time requirement

All participants remarked that the KK method was time-consuming and slow, whereas the FA280 analyzer was much faster and more efficient due to its high throughput.

*“Examining 200 samples using the KK method could take a week for just one person; it’s quite slow-paced.”*

*“Examining 200 samples with the fecal analyzer might take just over an hour.”*

*“The KK method requires more time; the FA280 takes shorter because the analyzer conducts tests on multiple fecal samples concurrently.”*

#### Smear examination

When examining the smears with the KK method, participants had to identify eggs by naked eyes and manually count the number. In contrast, the FA280 could automatically identify and capture images of eggs across more fields of view, and then record the results. However, the current analyzer was unable to perform automatic counting.

*“You have to manually count and calculate by drawing tally marks.”*

*“The analyzer is capable of automatically capturing images and recording.”*

*“The analyzer does not support automatic counting at present.”*

*“The FA280 offers multiple visual fields.”*

#### Requirement for technicians

In contrast to the FA280, the requirement for technicians of the KK method was much stricter. Participants expressed the need for professional training to become proficient in using the KK method. One participant added that the KK method also demanded higher qualifications from technical personnel, such as a solid background in medicine and adequate experience in the laboratory. Hence, those without lab experience require much longer training to master the KK method.

*“Using the KK method for fecal testing generally requires professional training.”*

*“Mastering the KK method typically takes about one day for individuals with prior laboratory testing experience, whereas it may require approximately a week for those without any prior experience in laboratory testing.”*

*“The KK method for smear examination demands stringent requirements on the technicians. It requires a wide-ranging professional knowledge, especially in the identification of different parasite eggs. In addition, sufficient experience and adequate training time are also important.”*

*“The fecal analyzer is fully automated. Whatever it sees, it automatically displays, identifies, and records. It’s more user-friendly.”*

Theme 2: differences in detecting results.

#### Accuracy

Two participants raised concerns about the potential for missed detections with the KK method. They believed that the fecal analyzer had a higher detection rate and provided more accurate results.

*“Using the KK method needs examining smears under a microscope, and sometimes adjusting the fine focus can lead to easily missing parasite eggs.”*

*“The fecal analyzer can detect a higher number of eggs compared to manual observation, leading to a higher detection rate.”*

*“I personally feel the machine is more accurate.”*

#### Involvement of human factor

Three participants agreed that the results obtained from the KK method relied on the subjective judgment of the technicians, and the capacity of human focus was limited. These factors could result in human errors. In contrast, the FA280 was less affected by human factors and required only manual calibration before initial use. Additionally, all participants mentioned that after the automatic identification by the fecal analyzer, results could be manually reviewed and adjusted.

*“The KK method relies on manual visual inspection, which is affected by subjective influences, while the fecal analyzer can automatically identify. When humans observe the smears, there’s always a possibility of fatigue and some human error.”*

*“But the fecal analyzer still needs calibration.”*

*“The results it produces need manual verification to confirm them. Sometimes false positives or false negatives may occur, so we have to manually review the images of the eggs it captures and check them with our naked eye.”*

#### Infection intensity

All participants stated that the KK method allowed for both qualitative and quantitative analysis, enabling infection intensity quantification. While the fecal analyzer could not yet quantify infection intensity, participants were confident that the establishment of standardized protocols could make it feasible.

*“The KK method allows for both qualitative and quantitative analysis.”*

*“It cannot calculate for quantitative analysis now (by the fecal analyzer).”*

Theme 3: differences in acceptance.

#### Acceptability

One participant mentioned that using the KK method was currently a last resort option, and all participants expressed a preference for using the fecal analyzer for stool examination.

*“Even though we may not fully accept the KK method, we have to acknowledge its importance, as it was the best available method in the past. Additionally, the World Health Organization recommends this method.”*

*“I'm willing to accept the fecal analyzer. I think it's excellent.”*

#### Future choice

All participants demonstrated their desire to utilize the FA280 in their future work. Moreover, one participant advocated for wider adoption of this technology.

*“In future work, I prefer to use the fecal analyzer.”*

*“I hope to promote the adoption of fecal analyzers, reducing the dependency on the KK method as the primary approach.”*

### Findings on the promotion of the FA280

The qualitative data from two medical institution administrators were categorized into three themes (Table 5): (i) advantages of promotion; (ii) challenges of promotion; and (iii) suggestions for promotion.

**Table 5:** Overview of themes and subthemes about the promotion of the FA280

Theme	Subtheme
Advantages of promotion	For medical institutions
	For population
Challenges of promotion	From fecal analyzer
	Public attitude
Suggestions for promotion	Scope of promotion
	Improvement for the fecal analyzer

Theme 1: advantages of promotion.

#### For medical institutions

All administrators agreed that the widespread use of the FA280 could significantly increase the quantity, efficiency, and quality of clonorchiasis detection. Moreover, one noted its potential financial benefits for lab medicine.

*“The fecal analyzer can increase the number of examinations conducted, and enhance the level of detection.”*

*“Clonorchiasis is just one aspect; the detection levels as well as the detection efficiency of other diseases requiring fecal examination can also be improved.”*

*“Indeed, it can also generate revenue for certain departments in hospitals, thereby providing them with financial benefits.”*

#### For population

An administrator pointed out that promoting the FA280 could increase the chances of diagnosing clonorchiasis in the population, thereby improving treatment opportunities.

*“Sometimes, clinical physicians may overlook the commonality of clonorchiasis and therefore do not suggest the relevant examinations. The FA280 can automatically identify components in feces, which increases the diagnostic chances for patients with this disease.”*

*“Accordingly, hospitals can provide treatment for individuals with positive test results.”*

Theme 2: challenges of promotion.

#### From fecal analyzer

One administrator expressed concerns about the lack of standardized calibration procedures for the FA280. Furthermore, the high cost of the analyzer and the absence of uniform pricing for patients’ testing services were highlighted by the other administrator.

*“The data isn’t shared at the moment; each machine is calibrated individually.”*

*“The entire instrument may be relatively expensive, costing several hundred thousand.”*

*“Currently, the challenge revolves around pricing for each testing service. There isn’t an appropriate pricing scheme. However, if a reasonable charge based on the cost is determined, the FA280 could be more easily promoted in the clinical practices and general hospitals.”*

#### Public attitude

An administrator also mentioned that the low awareness among the public regarding detecting and the insufficient attention from the government to clonorchiasis hindered the widespread adoption of the FA280.

*“There is a lack of awareness among the general population about clonorchiasis, leading to insufficient screening awareness.”*

*“The government does not pay enough attention to clonorchiasis.”*

Theme 3: Suggestions for promotion.

#### Scope of promotion

All administrators believed that the scope of FA280 promotion varied depending on the prevalence of clonorchiasis in different regions. Medical institutions in high-prevalence areas could commonly be equipped with the fecal analyzer, but in low-prevalence areas, only a limited number of comprehensive hospitals needed it.

*“In highly endemic areas with clonorchiasis like the Pearl River Delta, it would be better for medical institutions capable of professional examination for parasites to have at least one of this kind of analyzers.”*

*“In my opinion, comprehensive hospitals in areas with low prevalence should universally adopt this analyzer.”*

#### Improvement for the fecal analyzer

Due to the unavailability of shared calibration data for the analyzers at present, an administrator suggested the proposed unifying and sharing calibration standards. In addition, the importance of establishing standardized criteria for quantitative testing to determine the infection intensity was emphasized.

*“Theoretically, it’s feasible to share calibration data. Previously well-calibrated data obtained by sharing could be pre-loaded before the instrument leaves the factory. However, this requires cooperation with the manufacturer, and we have not yet achieved it.”*

*“For instance, we could fix the fecal weight in the sampling tubes. Then, for a total of 100 images captured, we could randomly select 20 images. The number of images containing C. sinensis eggs among these 20 could serve as a standardized measure of infection intensity.”*

## Discussion

In this study, a mixed method was applied, with the KK method as a control, to evaluate the performance, applicability, and feasibility for wider adoption of the FA280 in diagnosing clonorchiasis. In contrast to studies relying solely on quantitative or qualitative methods, the mixed method integrated insights gained from both above approaches, offering more comprehensive analysis and understanding [42, 43]. Furthermore, due to the lack of a gold standard method for fecal examination, other studies on the FA280 mostly considered the direct smear method, the FECT, or ELISA as the reference [28–30, 44–46]. However, given the extensive application and reliability of the KK method in large-scale epidemiological surveys of clonorchiasis in China, our study compared its results with those of the FA280.

The quantitative results showed that a highly accepted agreement between the FA280 and the KK method in *C. sinensis* detection $(\kappa = 0.82)$. Discrepancies in positive results between the two methods were mainly observed when infection intensity was extremely low (EPG < 24). These indicated that the performance of the FA280 was comparable to that of the KK method. Previous studies have shown that the accuracy of traditional microscopic stool examinations, such as the KK method, tended to decline when infection intensity was low [25]. As the FA280 was also based on microscopic stool examinations, it might also have a similar limitation. These potentially influenced the agreement rate of results from the two methods. Thus, developing more sensitive examination methods was necessary for stools with very low infection intensity. Notably, the FA280 also detected very low rates (< 0.5%) of other parasites (e.g., hookworm and pinworm), indicating its capacity to differentiate various parasite species. Given that these parasites were not endemic to the study area, our analysis focused solely on the detection performance of *C. sinensis*. Nevertheless, previous studies have confirmed its excellent differentiation capability for other parasitic eggs, and Peng et al. demonstrated the FA280’s comparable performance to FECT in hospital patients suspected of clonorchiasis [28–30, 44–46]. Our study was a supplement to their work, providing evidence that FA280 could be effectively applied in diagnosing clonorchiasis in endemic areas. As a result, all of the above demonstrated that the FA280 showed acceptable diagnosis performance.

The qualitative results illustrated the advantages of the FA280 over the KK method. The FA280 required minimal manual handling and processed multiple samples simultaneously, significantly reducing labor costs and testing time while enhancing efficiency and throughput. Its automatic analysis functions and more fields of view eliminated human errors and greatly improved the detection rate of *C. sinensis*, while its high-resolution imaging allowed for retrospective checks and confirmation to increase result accuracy. The closed system of the FA280 ensured technician safety and maintained a hygienic environment. Finally, the FA280 simplifies the learning and operation process for medical technicians, freeing them from the physical strain associated with traditional methods and boosting their motivation for fecal examinations. These qualitative findings directly provided users’ perception of the FA280, confirming its multiple benefits [28]. In addition, technicians showed a higher acceptance of the FA280 compared to the KK method and expressed a greater willingness to use the FA280 in their future work. Therefore, the FA280 and other more advanced fecal analyzers could be considered primary diagnostic tools for clonorchiasis and could be promoted in medical institutions.

Based on the findings of this study, the following suggestions were proposed to facilitate better use and promotion of the fecal analyzer like FA280: (i) For accuracy improvement, regular calibration supported by a shared network of calibration data should be implemented, along with the development of appropriate manual review and confirmation procedures and performance monitoring systems. (ii) The FA280 currently was unable to quantify infection intensity. Quantitative experiments, such as correlating the parasite burden in patients with the number of eggs captured from these analyzers, should be encouraged to establish standardized quantification methods. (iii) Health institutions in high-prevalence areas were recommended to be equipped with the FA280 or other advanced fecal analyzers to assist in the diagnosis of clonorchiasis or other parasites. (iv) Standardized pricing for diagnostic services using fecal analyzers should be developed to ensure affordability and accessibility for patients. (v) The government should pay more attention to the prevention and control of clonorchiasis and diffuse relevant knowledge to the public to raise awareness about detection and treatment.

There were several limitations of this study. First, owing to the limited availability of the FA280 at present, our study was confined to a relatively small region. Future studies could broaden the study area to gather more data for better assessment once the FA280 was going to be widely adopted. Second, the qualitative study involved a limited sample size, because only a small number of health service staff in the study area had used both detection methods. Nevertheless, we followed the information saturation principle, obtaining professional insights into the FA280’s applicability and scalability, and demonstrated that differences in detection, results, and acceptability between the two methods were evident. Although most opinions were anticipated, we well organized and summarized them through semi-structured interviews, reflecting the real perspectives of technicians and providing concrete evidence. We believe this was valuable for promoting the FA280. If it was widely adopted in the future, we would expand the scope of interviews to provide a more comprehensive evaluation of the instrument. Third, the FA280 could not quantify infection intensity currently. Future research should focus on conducting quantitative experiments to optimize this functionality.

## Conclusions

In conclusion, the automated fecal analyzer FA280 demonstrated comparable accuracy to the KK method, and outperformed in terms of operational ease and user acceptance, with fewer human errors, suggesting its great potential for widespread application in clonorchiasis diagnosis.

## Supplementary Information

Additional file 1: The Interview Guidelines for the semi-structured interviews

Additional file 2: Table S1. Themes, subthemes, codes, and sample quotes relative to differences between the FA280 and the KK method. Table S2. Themes, subthemes, codes, and sample quotes about promotion of the FA280. Table S3. Participant demographic details

Additional file 3: Text S1. The detail procedures about detection by the FA280. Fig. S1. The collection cube of FA280. Fig. S2. The sample detection of FA280. Fig. S3. The results output of FA280

## Data Availability

The data that support the findings of this study are not publicly available due to the containment of private information that could compromise research participant consent, but the anonymized data are available from the corresponding author YL on reasonable request.

## Abbreviations

KK: Kato-Katz
FECT: Formalin-ether concentration technique
ELISA: Enzyme-linked immunosorbent assay
*CI*: Confidence interval
EPG: Eggs per gram

## References

[1] Na BK, Pak JH, Hong SJ. *Clonorchis sinensis* and clonorchiasis. Acta Trop. 2020;203:105309. 31862466 10.1016/j.actatropica.2019.105309

[2] Qian MB, Chen YD, Yan F. Time to tackle clonorchiasis in China. Infect Dis Poverty. 2013;2(1):4. 23849773 10.1186/2049-9957-2-4 PMC3707093

[3] Deng ZH, Fang YY, Zhang QM, Mao Q, Pei FQ, Liu MR. The control of clonorchiasis in Guangdong province, southern China. Acta Trop. 2020;202: 105246. 31672488 10.1016/j.actatropica.2019.105246

[4] Jiang ZH, Wan XL, Lv GL, Zhang WW, Lin Y, Tang WQ, et al. High prevalence of *Clonorchis sinensis* infection in Guangxi, Southern China. Trop Med Health. 2021;49(1):6. 33461625 10.1186/s41182-021-00297-0 PMC7814618

[5] Xu CX, Wang X, Wang SY, Wang BH. Investigation of epidemic status of clonorchiasis sinensis in western regions of Jilin Province. Chin J Schistosomiasis Control. 2019;32(3):314–6 (in Chinese). 10.16250/j.32.1374.2018149 32468799

[6] Han S, Zhang X, Chen R, Wen J, Li Y, Shu J, et al. Trends in prevalence of clonorchiasis among patients in Heilongjiang province, Northeast China (2009–2012): implications for monitoring and control. PLoS ONE. 2013;8(11): e80173. 24260354 10.1371/journal.pone.0080173 PMC3833891

[7] Qian MB, Keiser J, Utzinger J, Zhou XN. Clonorchiasis and opisthorchiasis: epidemiology, transmission, clinical features, morbidity, diagnosis, treatment, and control. Clin Microbiol Rev. 2024;37(1): e0000923. 38169283 10.1128/cmr.00009-23 PMC10938900

[8] Qiao T, Ma RH, Luo XB, Luo ZL, Zheng PM. Cholecystolithiasis is associated with *Clonorchis sinensis* infection. PLoS ONE. 2012;7(8):e42471. 22905137 10.1371/journal.pone.0042471 PMC3414519

[9] Fürst T, Keiser J, Utzinger J. Global burden of human food-borne trematodiasis: a systematic review and meta-analysis. Lancet Infect Dis. 2012;12(3):210–21. 22108757 10.1016/S1473-3099(11)70294-8

[10] Qiao T, Ma RH, Luo ZL, Yang LQ, Luo XB, Zheng PM. *Clonorcis sinensis* eggs are associated with calcium carbonate gallbladder stones. Acta Trop. 2014;138:28–37. 24945791 10.1016/j.actatropica.2014.06.004

[11] Lun ZR, Gasser RB, Lai DH, Li AX, Zhu XQ, Yu XB, et al. Clonorchiasis: a key foodborne zoonosis in China. Lancet Infect Dis. 2005;5(1):31–41. 15620559 10.1016/S1473-3099(04)01252-6

[12] Han S, Zhang X, Wen J, Li Y, Shu J, Ling H, et al. A combination of the Kato-Katz methods and ELISA to improve the diagnosis of clonorchiasis in an endemic area, China. PLoS ONE. 2012;7(10): e46977. 23056547 10.1371/journal.pone.0046977 PMC3466177

[13] Wu W, Qian X, Huang Y, Hong Q. A review of the control of clonorchiasis sinensis and *Taenia solium* taeniasis/cysticercosis in China. Parasitol Res. 2012;111(5):1879–84. 23052782 10.1007/s00436-012-3152-y

[14] Tang ZL, Huang Y, Yu XB. Current status and perspectives of *Clonorchis sinensis* and clonorchiasis: epidemiology, pathogenesis, omics, prevention and control. Infect Dis Poverty. 2016;5(1):71. 27384714 10.1186/s40249-016-0166-1 PMC4933995

[15] Foodborne trematode infections. https://www.who.int/news-room/fact-sheets/detail/foodborne-trematode-infections. Accessed 10 Dec 2024.

[16] Qian MB, Utzinger J, Keiser J, Zhou XN. Clonorchiasis. Lancet. 2016;387(10020):800–10. 26299184 10.1016/S0140-6736(15)60313-0

[17] Qian MB, Zhou XN. Clonorchis sinensis. Trends Parasitol. 2021;37(11):1014–5. 34229953 10.1016/j.pt.2021.05.011

[18] Choi MH, Ge T, Yuan S, Hong ST. Correlation of egg counts of *Clonorchis sinensis* by three methods of fecal examination. Korean J Parasitol. 2005;43(3):115–7. 16192753 10.3347/kjp.2005.43.3.115 PMC2712011

[19] Qian MB, Yap P, Yang YC, Liang H, Jiang ZH, Li W, et al. Accuracy of the Kato-Katz method and formalin-ether concentration technique for the diagnosis of *Clonorchis sinensis*, and implication for assessing drug efficacy. Parasit Vectors. 2013;6(1):314. 24499644 10.1186/1756-3305-6-314 PMC3816101

[20] Li HM, Qian MB, Yang YC, Jiang ZH, Wei K, Chen JX, et al. Performance evaluation of existing immunoassays for *Clonorchis sinensis* infection in China. Parasit Vectors. 2018;11(1):35. 29334990 10.1186/s13071-018-2612-3 PMC5769360

[21] Zhu TJ, Chen YD, Qian MB, Zhu HH, Huang JL, Zhou CH, et al. Surveillance of clonorchiasis in China in 2016. Acta Trop. 2020;203: 105320. 31877282 10.1016/j.actatropica.2019.105320

[22] Chen YD, Zhou CH, Xu LQ. Analysis of the results of two nationwide surveys on *Clonorchis sinensis* infection in China. Biomed Environ Sci. 2012;25(2):163–6. 22998822 10.3967/0895-3988.2012.02.006

[23] Choi MH, Park SK, Li Z, Ji Z, Yu G, Feng Z, et al. Effect of control strategies on prevalence, incidence and re-infection of clonorchiasis in endemic areas of China. PLoS Negl Trop Dis. 2010;4(2): e601. 20169061 10.1371/journal.pntd.0000601 PMC2821909

[24] Aiadsakun P, Sriwimol W, Thongbun N, Rui-On B, Thiparaksaphan K, Phainuice C, et al. Comparison of the complete filtration method using an automated feces analyzer with three manual methods for stool examinations. J Microbiol Methods. 2022;192: 106394. 34919972 10.1016/j.mimet.2021.106394

[25] Hong ST, Choi MH, Kim CH, Chung BS, Ji Z. The Kato-Katz method is reliable for diagnosis of *Clonorchis sinensis* infection. Diagn Microbiol Infect Dis. 2003;47(1):345–7. 12967748 10.1016/s0732-8893(03)00113-5

[26] Lee YJ, Won EJ, Cho YC, Kim SH, Shin MG, Shin JH. Utility of an automatic vision-based examination system (AVE-562) for the detection of *Clonorchis sinensis* eggs in stool. Ann Lab Med. 2021;41(2):221–4. 33063684 10.3343/alm.2021.41.2.221 PMC7591289

[27] Liu ZH, Sun HC, Huang JH. Evaluation of improved automatic fecal analyzer for the detection of *Clonorchis sinensis* eggs. Capital Food Med. 2022;29(16):83–5 (in Chinese).

[28] Boonyong S, Hunnangkul S, Vijit S, Wattano S, Tantayapirak P, Loymek S, et al. High-throughput detection of parasites and ova in stool using the fully automatic digital feces analyzer, orienter model FA280. Parasit Vectors. 2024;17(1):13. 38185634 10.1186/s13071-023-06108-1 PMC10771706

[29] Peng LJ, Lin JH, Peng XM. Comparative analysis of the effect of FA280S automatic stool analyzer on the detection of early-stage clonorchiasis. Adv Clin Med. 2024;14(01):351–6 (in Chinese).

[30] Wang C, Wang J, Cai XJ, Cai ZM. Evaluation of clinical application of orienter F280 automatic fecal analyzer. Contemp Med. 2022;28(08):18–20 (in Chinese).

[31] New generation fully automatic digital feces analyzer. https://www.orienterglobal.com/pro/12/225.html. Accessed 20 Oct 2024.

[32] Qian MB, Chen YD, Zhu HH, Zhu TJ, Zhou CH, Zhou XN. Establishment and role of national clonorchiasis surveillance system in China. Chi J Epidemiol. 2018;39(11):1496–500 (in Chinese). 10.3760/cma.j.issn.0254-6450.2018.11.015 30462961

[33] Germeni E, Szabo S. Beyond clinical and cost-effectiveness: the contribution of qualitative research to health technology assessment. Int J Technol Assess Health Care. 2023;39(1): e23. 37092753 10.1017/S0266462323000211 PMC11570152

[34] Yilmaz K. Comparison of quantitative and qualitative research traditions: epistemological, theoretical, and methodological differences. Eur J Educ. 2013;48(2):311–25.

[35] Bannister-Tyrrell M, Meiqari L. Qualitative research in epidemiology: theoretical and methodological perspectives. Ann Epidemiol. 2020;49:27–35. 32711056 10.1016/j.annepidem.2020.07.008

[36] Introduction to Xinhui District. https://www.xinhui.gov.cn/zlxh/xhyl/xhjj/. Accessed 10 Dec 2024.

[37] Wang KY, Shu HF, Fang YY, Zeng QS, Song T. Data analysis of clonorchiasis surveillance in high endemic areas of Guangdong Province in 2016–2020. Chin J Parasitol Parasit Dis. 2022;40(05):629–34 (in Chinese).

[38] Sichuan Orienter Bioengineering Co. L. FA280 ai+ feces analyzer. https://crownkenya.com/shows/pdf/fa280.pdf. Accessed 20 Oct 2024.

[39] McHugh ML. Interrater reliability: the kappa statistic. Biochem Med (Zagreb). 2012;22(3):276–82. 23092060 PMC3900052

[40] Saunders B, Sim J, Kingstone T, Baker S, Waterfield J, Bartlam B, et al. Saturation in qualitative research: exploring its conceptualization and operationalization. Qual Quant. 2018;52(4):1893–907. 29937585 10.1007/s11135-017-0574-8 PMC5993836

[41] Braun V, Clarke V. Using thematic analysis in psychology. Qual Res Psychol. 2006;3(2):77–101.

[42] Farquhar MC, Ewing G, Booth S. Using mixed methods to develop and evaluate complex interventions in palliative care research. Palliat Med. 2011;25(8):748–57. 21807749 10.1177/0269216311417919

[43] Palinkas LA, Mendon SJ, Hamilton AB. Innovations in mixed methods evaluations. Annu Rev Public Health. 2019;40:423–42. 30633710 10.1146/annurev-publhealth-040218-044215 PMC6501787

[44] Chen YM, Zhang CY, Long TT, Deng SY, Xie H, He Y. Clinical application evaluation of WWT/FA280 automatic stool analyzer. J North Sichuan Med Coll. 2022;37(02):187–9 (in Chinese).

[45] Long TT, Chen YM, Tao Z, Jiang H. The clinical application analysis of automatic fecal analysis system FA280. Chin Med Devices. 2022;37(04):132–5 (in Chinese).

[46] Long TT, Deng SY, Zhang CY, Meng Q, Jiang H. Performances evaluation of FA280 fully automatic digital feces analyzer. Chin Med Equipment J. 2021;42(12):48–52 (in Chinese).

