# Supplementary material for: Assessment of the application of the FA280—a fully automated fecal analyzer for diagnosing clonorchiasis: a mixed-method study
Source: Infect Dis Poverty. 2025 Jan 6;14:1. doi: 10.1186/s40249-024-01271-8 (PMC11702166; doi:10.1186/s40249-024-01271-8)
Supplement: Supplementary file 1 — Additional file 1: The Interview Guidelines for the semi-structured interviews [file 40249_2024_1271_MOESM1_ESM.docx]

**Semi-structured Interview Questions**

| **The interviewer will record in notes** |
| --- |
| - Date of interview |
| - Time interview commences; time interview concludes |
| - Participant number |
| - Gender of participant |

| **Health service staff semi-structured interview** |
| --- |
| 1. What is your professional title? |
| 1. How many years of experience do you have in the prevention and control of clonorchiasis? |
| 1. How many years of experience do you have in fecal examination? |
| 1. Could you share your thoughts or feelings about your training or learning experience with the KK method? |
| 1. Could you share your thoughts or feelings about your training or learning experience with the FA280? |
| 1. Could you describe your feeling and experience with using the KK method for fecal examination? |
| 1. Could you describe your feeling and experience with using the FA280 for fecal examination? |
| 1. What do you think of the results obtained from the KK method? |
| 1. What do you think of the results obtained from the FA280? |
| 1. Do you have any further insights on the differences between these methods? |
| 1. Which type of fecal detection method do you prefer? |
| 1. Are you willing to use the fecal analyzer in future work? |

| **Medical institution managers semi-structured interview** |
| --- |
| 1. Do you think it can be promoted to medical institutions? |
| 1. Which level of medical institution do you think is better to set up this type of fecal analyzer? |
| 1. What do you think are the benefits of promoting it to medical institutions? |
| 1. What do you think are the challenges of promoting? |
