# Supplementary material for: Assessment of the application of the FA280—a fully automated fecal analyzer for diagnosing clonorchiasis: a mixed-method study
Source: Infect Dis Poverty. 2025 Jan 6;14:1. doi: 10.1186/s40249-024-01271-8 (PMC11702166; doi:10.1186/s40249-024-01271-8)
Supplement: Supplementary file 2 — Additional file 2: Table S1. Themes, subthemes, codes, and sample quotes relative to differences between the FA280 and the KK method. Table S2. Themes, subthemes, codes, and sample quotes about promotion of the FA280. Table S3. Participant demographic details [file 40249_2024_1271_MOESM2_ESM.docx]

**Themes, subthemes, codes, and sample quotes for qualitative data**

**Table S1. Themes, subthemes, codes, and sample quotes relative to differences between the FA280 and the KK method**

| Theme | Subtheme | Codes | Sample Quotes |
| --- | --- | --- | --- |
| Differences in testing procedures | Feeling about the testing process | KK: easy to learn  KK: cumbersome to operate  FA280: easy to learn  FA280: easy to operate | 1. *“The KK method is easy to learn.”* 2. *“The KK method is a bit cumbersome to operate.”* 3. *“Learning to use the fecal analyzer is much easier compared to the KK method.”* 4. *“The operation of the fecal analyzer is quite simple. Once the samples are collected, you place them directly into the instrument and let it do the rest.”* |
|  | Laboratory environment | KK: dirty and odorous  FA280: a little odorous  FA280: clean | 1. *“During the preparation of KK smears, there is an odor, and the environment is not pleasant, quite dirty, and smelly. Then, during the process of examining the smears, as it involves using a microscope and looking at them with the naked eye, there is still an odor, and the environment is not pleasant either.”* 2. *“Both the fecal analyzer and the KK method have odors, so how could there be no smell? However, the KK method has a stronger odor, while the* *instrument has less odor.”* 3. *“The environment is excellent, and using fecal analyzer is very clean.”* |
|  | Time requirement | KK: time-consuming, slowly  FA280: time-saving, rapidly  KK takes longer than FA280 | 1. *“Examining 200 samples using the KK method could take a week for just one person; it's quite slow-paced.”* 2. *“Examining 200 samples with the fecal analyzer might take just over an hour.”* 3. *“The KK method requires more time; the FA280 takes shorter because the analyzer conducts tests on multiple fecal samples concurrently.”* |
|  | Smear examination | KK: manually identify count and record  FA280: automatically identify and record  FA280: unable to count automatically  FA280: multiple visual fields | 1. *“You have to count and calculate manually by drawing tally marks.”* 2. *“**The analyzer is capable of automatically capturing images and recording.”* 3. *“**The analyzer does not support automatic counting at present.”* 4. *“**The FA280 offers multiple visual fields.”* |
|  | Requirement for technicians | KK: strict  FA280: user-friendly | 1. *“The KK method for smear examination demands stringent requirements on the technicians. It requires a wide-ranging professional knowledge, especially in the* *identification of different parasite eggs. In addition,* *sufficient* *experience and adequate training time are also important.”* 2. *“Using the KK method for fecal testing generally requires professional training.”* 3. *“Mastering the KK method typically takes about one day for individuals with prior laboratory testing experience, whereas it may require approximately a week for those without any prior experience in laboratory testing.”* 4. *“The fecal analyzer is fully automated. Whatever it sees, it automatically displays, identifies, and records. It's more user-friendly.”* |
| Differences in detecting results | Accuracy | KK: high rate of missed detection  FA280: higher detection rate  FA280: more accurate | 1. *“Using the KK method needs examining smears under a microscope, and sometimes adjusting the fine focus can lead to easily missing parasite eggs.”* 2. *“The fecal analyzer can detect a higher number of eggs compared to manual observation, leading to a higher detection rate.”* 3. *“I personally feel the machine is more accurate.”* |
|  | Involvement of human factor | KK: subjective judgment  FA280: manual calibration before initial use  FA280: manual verification | 1. *“The KK method relies on manual visual inspection, which is affected by subjective influences, while the fecal analyzer can automatically identify. When humans observe the smears, there's always a possibility of fatigue and some human error.”* 2. *“But the fecal analyzer still needs calibration.”* 3. *“The results it produces need manual verification to confirm them. Sometimes false positives or false negatives may occur, so we have to manually review the images of the eggs it captures and check them with our naked eye.* |
|  | Infection intensity | KK: allow for quantifying  FA280: unable to quantify | 1. *“The KK method allows for both qualitative and quantitative analysis.”* 2. *“**It cannot calculate for quantitative analysis now”* |
| Differences in acceptance | Acceptability | KK: no choice but to accept  FA280: willing to accept | 1. *“Even though we may not fully accept the KK method, we have had to acknowledge its importance, as it was the best available method in the past. Additionally, the World Health Organization recommends this method.”* 2. *“I'm willing to accept the fecal analyzer. I think it's excellent.”* |
|  | Future choice | Use FA280  Promote FA280 | 1. *“In future work, I prefer to use the fecal analyzer.”* 2. *“I hope to promote the adoption of fecal analyzers, reducing the dependency on the KK method as the primary approach.”* |

**Table S2. Themes, subthemes, codes, and sample quotes about the promotion of the FA280**

| Theme | Subtheme | Codes | Sample Quotes |
| --- | --- | --- | --- |
| Advantages of promotion | For medical institutions | increase detection amount  increase detection level  increase detection efficiency  generate revenue | 1. *“The fecal analyzer can increase the number of examinations conducted, and enhance the level of detection.”* 2. *“Clonorchiasis is just one aspect; the detection levels as well as the detection efficiency of other diseases requiring fecal examination can also be improved.”* 3. *“Indeed, it can also generate revenue for certain departments in hospitals, thereby providing them with financial benefits.”* |
|  | For population | increase the diagnostic chances  improve treatment opportunities | 1. *“Sometimes, clinical physicians may overlook the commonality of clonorchiasis and therefore do not suggest the relevant examinations. The FA280 can automatically identify components in feces, which increases the diagnostic chances for patients with this disease.”* 2. *“Accordingly, hospitals can provide treatment for individuals with positive test results.”* |
| Challenges of promotion | From fecal analyzer | No uniform calibration standard  High cost  No uniform pricing for patients | 1. *“The data isn’t shared at the moment; each machine is calibrated individually.”* 2. *“The entire instrument may be relatively expensive, costing several hundred thousand.”* 3. *“Currently, the challenge revolves around pricing for each testing service. There isn't an appropriate pricing scheme. However, if a reasonable charge based on the cost is determined, the FA280 could be more easily promoted in the clinical practices and general hospitals.”* |
|  | Public attitude | Public: lack of awareness  Government: neglect | 1. *“There is a lack of awareness among the general population about clonorchiasis, leading to insufficient screening awareness.”* 2. *“The government does not pay enough attention to clonorchiasis.”* |
| Suggestions for promotion | Scope of promotion | Areas with high prevalence  Areas with low prevalence | 1. *“In highly endemic areas with clonorchiasis like the Pearl River Delta, it would be better for medical institutions capable of professional examination for parasites to have at least one of this kind of analyzer.”* 2. *“In my opinion, comprehensive hospitals in* *areas with low prevalence should universally adopt this analyzer.”* |
|  | Improvement for the fecal analyzer | Share calibration standard  Standardized protocol for quantifying infection intensity | 1. *“Theoretically, it's feasible to share calibration data.* *Previously well-calibrated data* *obtained by sharing could be pre-loaded before* *the instrument leaves the factory. However, this requires cooperation with the manufacturer, and we have not yet achieved it.”* 2. *“For instance, we could fix the fecal weight in the sampling tubes. Then, for a total of 100 images captured, we could randomly select 20 images. The number of images containing C. sinensis eggs among these 20 could serve as a standardized measure of infection intensity.”* |

**Demographic details of interviews**

**Table S3. Participant demographic details**

| Participant | Gender | Professional title | Experience for control of clonorchiasis (years) | Experience for fecal examination (years) |
| --- | --- | --- | --- | --- |
| A | Female | Associate chief technician | 20 | 20 |
| B | Female | Junior technician | 5 | 5 |
| C | Female | Chief physician | 36 | 36 |
