# Supplementary material for: Assessment of the application of the FA280—a fully automated fecal analyzer for diagnosing clonorchiasis: a mixed-method study
Source: Infect Dis Poverty. 2025 Jan 6;14:1. doi: 10.1186/s40249-024-01271-8 (PMC11702166; doi:10.1186/s40249-024-01271-8)
Supplement: Supplementary file 3 — Additional file 3: Text S1. The detail procedures about detection by the FA280. Fig. S1. The collection cube of FA280. Fig. S2. The sample detection of FA280. Fig. S3. The results output of FA280 [file 40249_2024_1271_MOESM3_ESM.docx]

**Text S1. The detail procedures about detection by the FA280**

The FA280 fully automated fecal analyzer employed automatic sedimentation and concentration technology for detection. (i) Sample collection and sending. Approximately 0.5 g of a fecal sample was collected in a filtered sample collection tube and submitted for testing [1]. The collection tube contains a mesh structure, which effectively concentrates parasite eggs. The sample tube sending utilized a track-type sample carrier. (ii) Microscopic observation. The device initiated microscopic observation, acquiring images and recording attributes like color, shape, and consistency. (iii) Intelligent dilution. By detecting the sample turbidity, the diluent was automatically injected into the sample tube, followed by intelligent dilution to reach standard concentration, thereby avoiding invalid results in microscope images due to excessive concentration or overly thick samples. (iv) High-frequency pneumatic mixing. High-frequency pneumatic mixing was employed to fully blend the diluent with the fecal sample, preserving the integrity of solid components and aiding in the filtration of non-pathological residues. (v) Multi-field tomographic imaging. Through the multi-field tomography and the automatic locating tracking technology, the microscope of the FA280 automatically switched between low magnification (×10) and high magnification (×40) to locate and identify parasite eggs and capture high-resolution images. Meanwhile, the magnified view function could present specific structures of parasites egg, such as egg caps and acromion. (vi) Result reports. The images were then analyzed by the software to generate a result report on the screen. The report could present a list of sample indicators, including color, physical characteristics, and the presence of various pathological elements such as RBC, WBC, and different parasitic species like *C. sinensis*. Besides, the report also showed magnified images of a stool sample with visible parasite structures, based on which, results were adjusted upon manual image review.

1. Sichuan Orienter Bioengineering Co. L. New generation fully automatic digital feces analyzer. <https://www.orienterglobal.com/pro/12/225.html> (Updated on October 2024).


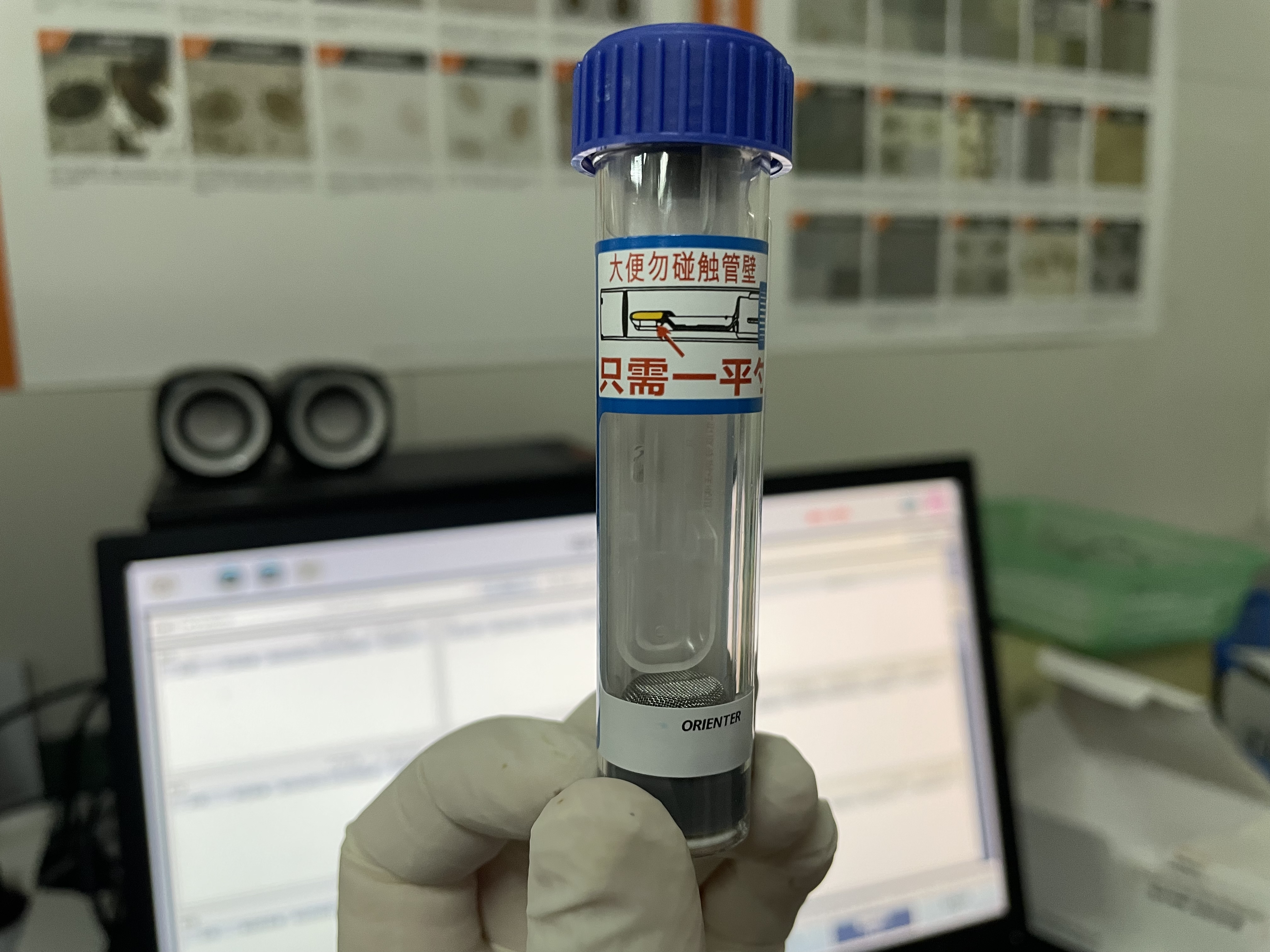


**Fig. S1.** The collection cube of FA280.


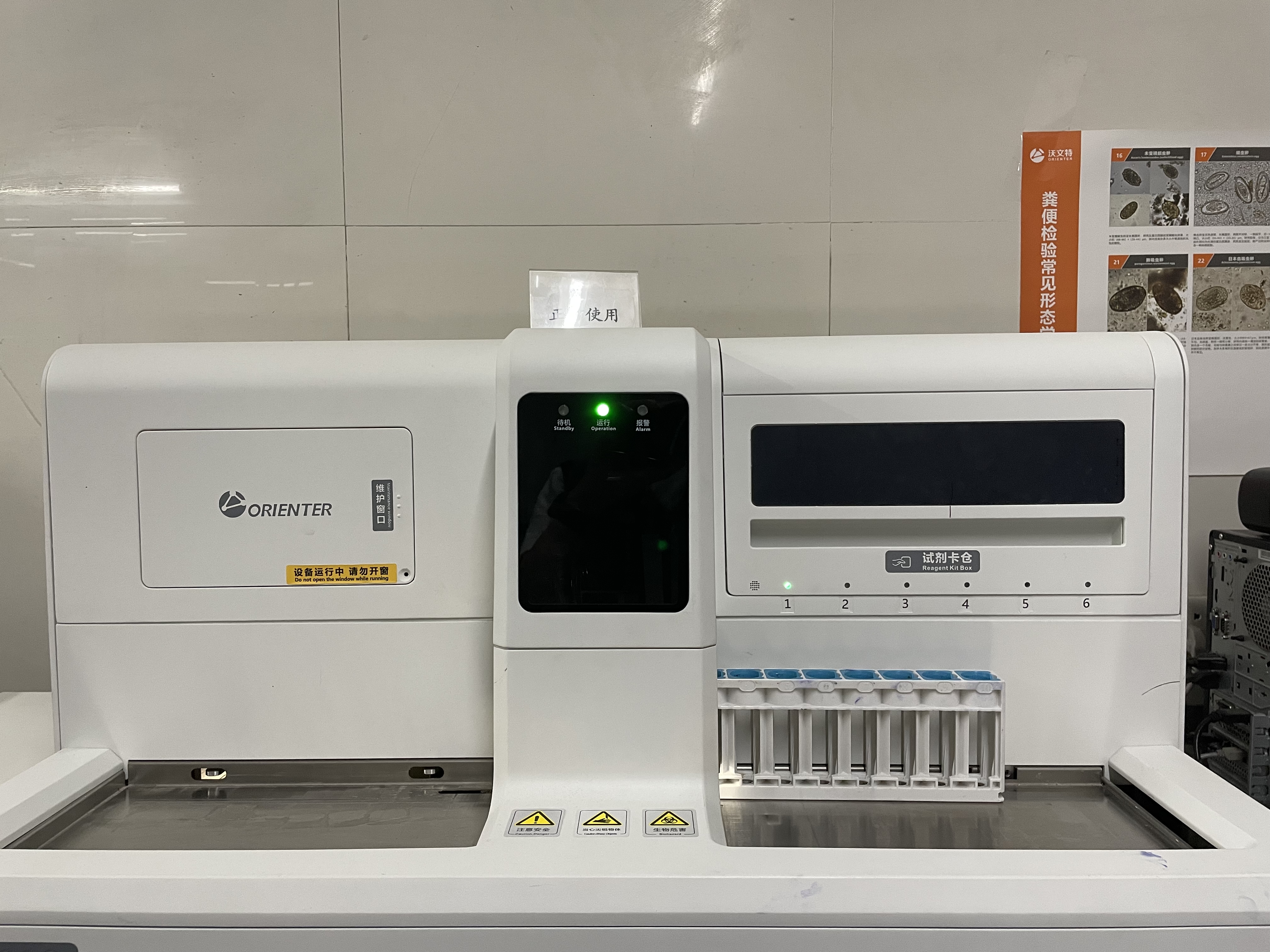


**Fig. S2.** The sample detection of FA280.


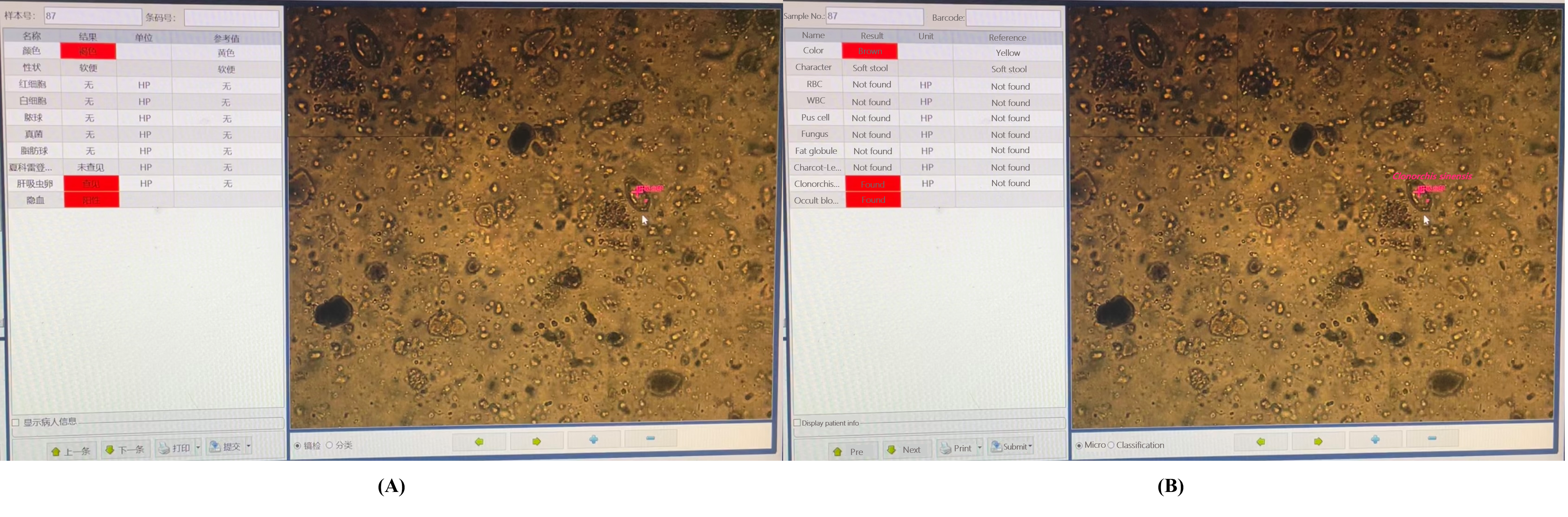


**Fig. S3.** The results output of FA280. (A) was the original one, and (B) was the translated one.
